# Epithelial Barrier Dysfunction in Chronic Respiratory Diseases

**DOI:** 10.3389/fphys.2021.691227

**Published:** 2021-06-24

**Authors:** François M. Carlier, Charlotte de Fays, Charles Pilette

**Affiliations:** ^1^Pole of Pneumology, ENT, and Dermatology, Institute of Experimental and Clinical Research, Université catholique de Louvain, Brussels, Belgium; ^2^Department of Pneumology and Lung Transplant, Centre Hospitalier Universitaire UCL Namur, Yvoir, Belgium; ^3^Department of Pneumology, Cliniques universitaires St-Luc, Brussels, Belgium

**Keywords:** barrier dysfunction, airway epithelium, mucosal immunity, COPD, asthma, cystic fibrosis, idiopathic pulmonary fibrosis

## Abstract

Mucosal surfaces are lined by epithelial cells, which provide a complex and adaptive module that ensures first-line defense against external toxics, irritants, antigens, and pathogens. The underlying mechanisms of host protection encompass multiple physical, chemical, and immune pathways. In the lung, inhaled agents continually challenge the airway epithelial barrier, which is altered in chronic diseases such as chronic obstructive pulmonary disease, asthma, cystic fibrosis, or pulmonary fibrosis. In this review, we describe the epithelial barrier abnormalities that are observed in such disorders and summarize current knowledge on the mechanisms driving impaired barrier function, which could represent targets of future therapeutic approaches.

## Introduction

The respiratory epithelium is in direct contact with the external environment, which includes noxious stimuli such as microbes, allergens, cigarette smoke (CS), and other air pollutants such as ozone or particulate matter (PM). In order to provide an effective protection to airways integrity, numerous distinct mechanisms are normally minutely coordinated. However, the actors of the epithelial barrier may become altered in respiratory diseases such as chronic obstructive pulmonary disease (COPD), asthma, cystic fibrosis (CF), and idiopathic pulmonary fibrosis (IPF), following the causative and/or other pathogenic mechanisms. This results, in turn, in further damage due to defective frontline defense against inhaled agents, which may lead to inflammatory or infectious exacerbations.

In this review, we focus on the specific alterations of the epithelial barrier in chronic diseases of the lungs and their underlying mechanisms, which could be useful to identify innovative preventive or therapeutic targets aiming to maintain lung barrier integrity.

## Experimental Tools to Assess Epithelial Functions

To help the reader apprehend how the biology and functions of the airway epithelium (AE) have been assessed by researchers in the last decades, we provide hereunder a summary of the main basic concepts and related techniques.

### Mucociliary Clearance

Mucociliary clearance constitutes an active defense mechanism, ensuring the continuous evacuation of inhaled particles trapped in mucus. As it relies on both mucus viscosity and ciliary function, the assessment of mucociliary clearance mainly relates to parameters associated to these factors. Mucus viscosity depends on the composition and hydration of its components. The analysis of mucins (mainly MUC5AC and MUC5B), on the one hand, and of hydro-ionic fluxes, on the other hand, provides valuable information on mucus quality. In addition, rheological analysis directly assesses the physical flow properties of mucus (Fahy and Dickey, 2010; Wagner et al., 2018; Ma et al., 2018). Ciliary function can also be measured by dynamic studies (such as cilia beating frequency or propel mucus speed), which may then be correlated with structural features such as length of cilia and number of cilia per cell (Tilley et al., 2015).

### Physical Barrier Function, Apical Complexes

The AE constitutes a physical barrier, preventing the trespassing of inhaled particles. Transepithelial electrical resistance (TER) consists of the measurement of electric resistance across a cellular layer [such as AE cells redifferentiated in air/liquid interface (ALI)] and somehow provides a quantification of the integrity of the epithelial barrier (Srinivasan et al., 2015). This measure reflects the functionality of cell–cell adhesion, mainly provided by apical junctional complexes (AJC) (Powell, 1981). An indirect measure of the relative impermeability of the AE consists of measuring the (absence of) passage of stained, high-molecular weight molecules such as fluorescein isothiocyanate (FITC)-labeled dextran. Surrogate measures of the barrier function also frequently include gene and protein expression of AJC components, such as occludin (OCLN), claudins (CLDNs), tight junction protein-1/zonula occludens-1 (TJP1), junctional adhesion molecules (JAMs), E-cadherin (ECAD), and β-catenin (CTNNB1).

### Epithelial Polarity and Transcytosis

Exposed to luminal air and basolateral interstitium (lamina propria), the AE acts as an interface between outer and inner conditions, a function that requires adequate polarization of its cells. The assessment of AJC function and integrity (see above) gives relevant information on the polarization of the epithelium, as AJC physically separate the lumen from the basoapical pole but have also been shown to activate pathways promoting epithelial polarity (Matter and Balda, 2003; McCrea et al., 2009). Polarity can also be assessed on a more functional basis. As an example, the transcytosis of dimeric (d-) immunoglobulin (Ig) A depends on the transcellular basoapical routing of its dedicated receptor, the polymeric Ig receptor (pIgR). The cleavage of this receptor at the apical side of the epithelium releases its extracellular part, called secretory component (SC), either free or bound to dimeric IgA, thereby generating secretory (S-)IgA (Carlier et al., 2016). Both pIgR expression levels and apical release of SC or S-IgA witness epithelial polarization (Pilette et al., 2001a) and may be assessed both *in vivo* [in bronchoalvelolar lavage fluid (BALF) and sputum] and *in vitro* in ALI-redifferentiated AE. The transport of other molecules (e.g., proteins, ions) across the epithelium may also be used to evaluate apico-basal or basoapical polarization of the AE.

### Cell Differentiation

The AE is composed by numerous cell types, including basal, goblet, ciliated, club, and neuroendocrine cells, along with recently identified ionocytes (Travaglini et al., 2020). Their relative numbers vary along the respiratory tract, and their proportion is altered in chronic respiratory diseases. The identification and quantification of these cells may be assessed by targeting specific protein markers or transcription factors, as recapitulated in Table 1.

**TABLE 1:** Main cell types composing the AE and associated protein and transcription factors (non-exhaustive list) reported to be specific of each cell type within the AE.

Cell type	Commonly used markers	Transcription factor	References
Basal cells	KRT5, KRT14	TP63	Rock et al., 2010
Goblet cells	Mucins (MUC5AC, MUC5B)	SPDEF, FOXA3	Park et al., 2007; Chen et al., 2014; Knoop and Newberry, 2018
Ciliated cells	β-tubulin IV, acylated tubulin	FOXJ1, RFX2, MCIDAS	You et al., 2004; Bisgrove et al., 2012; Whitsett, 2018
Club cells	SCGB1A1	FOXM1	Ustiyan et al., 2012; Hiemstra and Bourdin, 2014; Zuo et al., 2018
Neuroendocrine cells	Serotonin, chromogranin	ASCL1	Borges et al., 1997; Branchfield et al., 2016
Ionocytes	CFTR*	FOXI1	Montoro et al., 2018; Plasschaert et al., 2018

*CFTR is not properly specific of ionocytes, but ionocytes have been isolated based on their enrichment in CFTR.

## Epithelial Alterations in Chronic Respiratory Diseases

**FIGURE 1:**
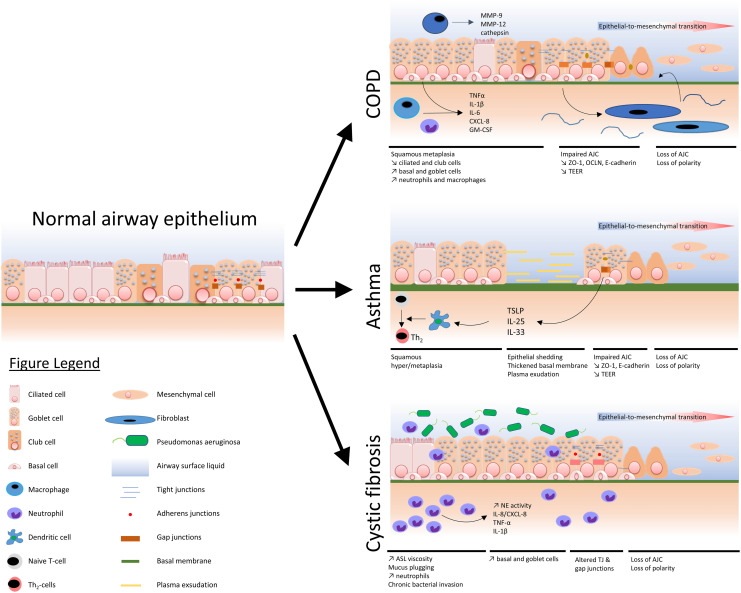
Schematized view of main epithelial alterations observed in COPD, asthma, and cystic fibrosis as compared with a normal airway epithelium.

A comprehensive view of the main airway epithelium alterations displayed in COPD, asthma, and CF is depicted in Figure 1.

### COPD

COPD constitutes the third leading cause of death worldwide (World Health Organization, 2016; Vogelmeier et al., 2017). It is a frequent, preventable but poorly treatable disease characterized by a progressive and mostly irreversible airflow obstruction that is due to a combination of small airway disease with obstruction and disappearance of bronchioles and emphysema with the destruction of alveolar walls, ultimately leading to respiratory failure (Vogelmeier et al., 2017). Its major cause is cigarette smoking, although genetic predisposition and other toxic exposures (air pollution, occupational, biomass) also play a role (Salvi and Barnes, 2009). As CS contains more than 7,000 chemicals, including oxidative gases, heavy metals, and carcinogenic substances, the load of toxic particles is especially high in smokers (U.S. Department of Health and Human Services, 2014), among which approximately 25% will develop COPD. The AE structure and biology are profoundly altered in COPD, displaying major changes in terms of barrier structure, cell differentiation, inflammation, and cell polarity, all of which may affect its salient functions.

The cell composition of the pseudostratified respiratory epithelium is deeply modified in COPD, with basal cell hyperplasia historically considered as one of the earliest change associated with cigarette smoking (Auerbach et al., 1961). The normal human AE includes ciliated cells (50–90% of cells), secretory cells that are mainly goblet cells in large airways and club cells in small airways, basal cells representing airway progenitors, as well as rare subsets including neuroendocrine cells, ionocytes, and tuft cells (Montoro et al., 2018). Recent studies showed that in COPD, basal cells exhibit impaired stemness (Staudt et al., 2014; Ghosh et al., 2018) and altered transcriptional programming (Hackett et al., 2011; Crystal, 2014). Second, and similarly to asthma, COPD patients display goblet cell hyperplasia (in large airways) and metaplasia (in small airways, where virtually no goblet cells are found in healthy subjects) (Kim et al., 2008; Gohy et al., 2019), a feature that is driven by the transcription factors SPDEF and FOXA3 (Park et al., 2007; Chen et al., 2014). This secretory feature is possibly more directly related to smoking rather to COPD *per se*, as it is already observed in smokers without functional evidence of the disease, i.e., with normal lung function (Saetta et al., 2000). Third, decreased numbers of ciliated cells are observed in the COPD AE (Jeffery, 2000; Gohy et al., 2019), with remaining cells exhibiting dysfunctional primary cilia (Perotin et al., 2018), reduced cilia beating frequency (Yaghi et al., 2012; Yaghi and Dolovich, 2016), and cilia shortening (Ballenger, 1960; Leopold et al., 2009). Finally, club cells numbers and their production of secretoglobulin family 1A member 1 (SCGB1A1) are decreased in the COPD (Pilette et al., 2001b; Gamez et al., 2015). Interestingly, the altered lineage differentiation of the AE observed in COPD lung tissue may be recapitulated *in vitro* following reconstitution of the AE by culture in ALI of primary human bronchial epithelial cells (HBEC) from COPD patients (Carlier et al., 2018; Gohy et al., 2019), suggesting some form of “epithelial memory” of the disease that remains imprinted in the epithelium (Carlier et al., 2021), probably through epigenetic marks.

Epithelial-to-mesenchymal transition (EMT) is a physiological and dynamic process during which epithelial cells lose their polarity, adhesiveness, and anchorage to the basal membrane and acquire mesenchymal features, such as migratory abilities through the reorganization of their cytoskeleton (Kalluri and Weinberg, 2009; Lamouille et al., 2014). EMT programming is regulated by an intricate network of pathways, including Wingless/Integrase-1 (WNT) and transforming growth factor (TGF)-β (Lamouille et al., 2014). In the last decade, several studies showed that EMT is present in airway tissues from smokers and is further enhanced in COPD patients, both in small (Milara et al., 2013; Mahmood et al., 2015) and large airways (Sohal et al., 2010, 2011; Mahmood et al., 2015; Carlier et al., 2020). Thus, Sohal and colleagues observed increased expression of EMT-related S100A4 and matrix metalloproteinase (MMP)-9 in the large airways from COPD patients as compared with non-COPD smokers and non-smokers (Sohal et al., 2010, 2011). They showed that EMT also occurred in small airways in COPD, with increased S100A4 and vimentin protein expression (Mahmood et al., 2015). In parallel, Milara and colleagues found increased α-smooth muscle actin (α-SMA), vimentin, collagen type I, and decreased zonula occludens (ZO)-1 expression, both in small airways sections and in primary ALI-HBEC derived from smokers and COPD patients, as compared with non-smokers (Milara et al., 2013). In addition, phosphorylated (p) extracellular signal-regulated kinase (ERK) 1/2 and p-SMAD3 protein levels were similarly increased, strengthening the evidence that EMT occurs in the COPD (and smokers) AE. Interestingly, the dysregulation of these genes and pathways appears usually aggravated in COPD as compared with non-COPD smokers. Finally, our team recently showed that WNT/β-catenin signaling pathway is activated in the large airways of COPD patients, therefore contributing to enhance EMT, as *in vitro* extrinsic activation of WNT resulted in increased vimentin expression, fibronectin release, and p-SMAD2/TGF-β signaling (Carlier et al., 2020). EMT, associated with CS-induced TGF-β production, could therefore promote airway fibrosis, impaired epithelial repair, as well as carcinogenesis and metastasis potential (Bartis et al., 2014; Sohal, 2015; Jolly et al., 2018). As for lineage abnormalities, ALI cultures from COPD patients spontaneously reproduce mesenchymal features (Carlier et al., 2021; Milara et al., 2013), while CS may induce EMT in control HBEC, possibly as a result of TGF-β signaling (Gohy et al., 2015).

Squamous metaplasia consists of a histological lesion characterized by the reversible replacement of the normal columnar epithelium by flattened squamous cells (Puchelle et al., 2006). It is a common alteration of the AE in smokers, and it is associated with airway obstruction in COPD (Cosio et al., 1978; Araya et al., 2007). The role of TGF-β signaling in the generation of squamous metaplasia in COPD has been recently enlightened, as bronchial epithelial cells exposed to TGF-β display increased expression of involucrin, a terminal marker of squamous metaplasia (Gohy et al., 2019). In parallel, squamous metaplasia is also driven by epidermal growth factor (EGF)-induced EGF receptor (EGFR) downstream signaling (Shaykhiev et al., 2013).

Inflammation is a paramount feature in COPD that is thought to represent a major player in the disease pathophysiology (Barnes, 2016). Epithelial cells are indeed activated by CS and/or other inhaled irritants to produce inflammatory mediators such as tumor necrosis factor (TNF)-α, interleukin (IL)-1β, IL-6, IL-8/C-X-C motif chemokine ligand (CXCL)-8, or granulocyte-macrophage colony-stimulating factor (GM-CSF) (Gao et al., 2015). In addition, activated alveolar macrophages and recruited neutrophils secrete various cytokines and chemokines (including IL-8/CXCL-8). TNF-α, IL-1β, IL-6, and CXCL-8/IL-8 increased levels were observed in BALF from smokers (Kuschner et al., 1996) and in sputum from smokers and stable COPD patients (Keatings et al., 1996). IL-6 and CXCL-8/IL-8 levels were further increased in sputum during exacerbations of COPD patients (Wedzicha et al., 2000). In addition, several other cyto-/chemokines are increased in COPD such as growth-regulated oncogene (GRO)-α, monocyte chemoattractant protein (MCP)-1, IL-5, and IL-13. In parallel, neutrophils are increased in blood (Gunay et al., 2014), BALF (Lacoste et al., 1993; Traves et al., 2002), and sputum (Keatings et al., 1996; Pesci et al., 1998; Leckie et al., 2003) from COPD patients, probably as a result of enhanced CXCL-8/IL-8 release from activated epithelial cells and alveolar macrophages. In addition, the total number of macrophages in the airway lumen (Kuschner et al., 1996; Kaku et al., 2014) and in the BALF (Lacoste et al., 1993; Traves et al., 2002; Profita et al., 2010) is increased in smokers and COPD patients, likely playing a role in emphysema by releasing elastolytic enzymes such as MMP-9, elastase (MMP-12), and cathepsins (Russell et al., 2002).

Physical barrier function, provided by AJC (see below) is deteriorated in COPD. Indeed, zonula occludens proteins ZO-1 and OCLN are decreased both *in situ* in the native AE from COPD patients and *in vitro* in derived ALI-cultured epithelium, as compared with those from smokers (Carlier et al., 2021; Heijink et al., 2014). Similarly, the expression of E-cadherin, a central protein of adherens junctions, is also reduced in the COPD AE (Oldenburger et al., 2014). These descriptive data depict altered junctional complexes in COPD, suggesting impaired barrier function and increased epithelial permeability.

Finally, the polarization is altered in the COPD AE, following disruption of pIgR, which assumes the transport of polymeric Ig, i.e., dimeric IgA and IgM, into mucosal secretions. The pIgR/SC system is defective in the COPD AE, both *in situ* and *in vitro* in COPD-derived ALI-reconstituted AE (Carlier et al., 2021; Gohy et al., 2014), and local S-IgA deficiency in small airways was associated with epithelial inflammation and remodeling (Polosukhin et al., 2011, 2017). In addition, pIgR^–/–^ mice are more susceptible to develop airway fibrosis and emphysema upon aging (Richmond et al., 2016), indicating that altered pIgR expression could play a driving role in this disease. Finally, low serum IgA levels are associated with increased risk for COPD exacerbations (Putcha et al., 2018), while Ig replacement therapy in COPD patients with (IgG) antibody deficiency reduces the exacerbation rate and related hospitalizations (McCullagh et al., 2017), underlining the crucial role of infections and impaired local mucosal immunity in the pathogenesis of COPD exacerbations.

### Asthma

Asthma is an immune-mediated and epithelial disease that includes airway hyperreactivity, with a prevalence ranging from 1 to 22% of the population according to the countries (Masoli et al., 2004). It is most frequently associated with a type 2 (T2) immuno-inflammatory phenotype (Wenzel, 2012), while different subsets can be distinguished according to age of onset, allergic background, and comorbidities (obesity, nasal disease, aspirin sensitivity). A “non-T2” phenotype also exists but remains poorly understood. Common symptoms of asthma include episodes of cough, shortness of breath, and/or wheezing. Early onset, allergic asthma usually develops before the age of 16, following allergic sensitization to common inhaled allergens such as house dust mite [HDM, the main aeroallergen involved in allergic asthma (Gregory and Lloyd, 2011)].

Dysregulated epithelial barrier function is a central element in the pathogenesis of this prototype of asthma, since it facilitates translocation of inhaled allergens and viruses, which activate the type 2 immune signals from the epithelium and immune cells (Holgate Stephen et al., 2009). Thus, alteration of the barrier integrity is a major hallmark of asthma. Epithelial shedding has been widely reported in biopsies from asthma patients, particularly with mild disease (Sumi and Hamid, 2007). Although initially controversial, as it was attributed to artifactual per-procedure damage (OrdoÑEz et al., 2000), this feature was later supported by a more important degree of epithelium loss in asthma than in controls (Ollerenshaw and Woolcock, 1992). However, despite this local epithelial shedding, *in vivo* studies suggest that airway permeability to inhaled molecules remains unaltered (Elwood et al., 1983), possibly due to exudation of plasma fluid into the bronchial lumen (Dunnill, 1960). Indeed, based on animal studies, this phenomenon could cover the denudated basement membrane by a fibrin–fibronectin gel, which promotes epithelial repair and regeneration, notably through the binding of blood leukocytes (Erjefält et al., 1995; ErjefÄLt et al., 1997).

Epithelial AJCs, both witnessing and driving apico-basal polarization, are altered in asthma, with biopsies from asthma patients displaying abnormal patchy staining for tight junctions (TJ) proteins OCLN and ZO-1 (Xiao et al., 2011). Cultures of airway epithelial cells issued from asthma patients also revealed disruption of TJ proteins, as compared with control subjects (Xiao et al., 2011), while expression of adherens junctional proteins α-catenin and E-cadherin is also decreased (de Boer et al., 2008). In addition, *in vitro* studies provide evidence that various inhaled irritants, including allergens, viruses, and CS alter AJC (Wan et al., 1999; Olivera et al., 2007; Short et al., 2016). In this context, genome-wide association studies unraveled that the genetic susceptibility to develop asthma is partly related to barrier dysfunction, with identified polymorphisms in the cadherin-related family member 3 (CDHR3) gene. CDHR3 is a transmembrane protein, and the only receptor for rhinovirus (RV)-C (while other RVs bind to ICAM-1 and LDLR) (Bochkov et al., 2015; Basnet et al., 2019), and CDHR3 polymorphisms in asthma may result in increased RV-C epithelial trespassing. RV increases neutrophilia during virus-induced exacerbations of allergic asthma, leading to the release of neutrophil extracellular traps (NETs) in the so-called “NETosis” process. NETosis mediates direct cytotoxicity against epithelial cells, and inhibition of NETosis protects mice from type-2 immune response of allergic asthma (Toussaint et al., 2017), indicating that the epithelium also participates to mechanisms of viral-induced exacerbations of asthma. In addition, experimental murine models reproducing different asthma phenotypes (i.e., T2-eosinophilic, mixed, and non-T2 neutrophilic) were used to distinguish phenotype-related effects on epithelial barrier disruption, including TJ proteins and mucins. While several TJ proteins, such as ZO-1 and claudin-18, are downregulated in all asthma phenotypes (although in variable extents), claudin-4 overexpression is exclusively found in non-T2 (neutrophilic) asthma. Phenotype-related differences are also observed in mucins, as MUC5AC and MUC5B overexpression is present in all asthma phenotypes but with enhanced increases in mixed and neutrophilic asthma (Tan et al., 2019).

Asthma is also characterized by changes in cell composition of the epithelium, with a prominent goblet cell hyperplasia (in large airways) and metaplasia (in small airways), as previously described in COPD (Shimura et al., 1996; OrdoÑEz et al., 2001). Coupled with the hypertrophy of submucosal glands, it results in an excessive production of altered mucus, enriched in MUC5AC. This activation of mucus secretion occurs following EGFR activation, which is associated with the presence of an MUC5B (preferentially a low charge, slow migrating form on the basis of its electrophoresis mobility) that is normally not found in healthy mucus (Welsh et al., 2017) as well as of insoluble MUC2 (Thornton et al., 1997; Sheehan et al., 1999; Morcillo and Cortijo, 2006; Bonser and Erle, 2017) that is normally present only in very small amounts (Thornton et al., 2008).

EMT has been described in asthma (Liu et al., 2017) and has been shown to be induced *in vitro* by exposing mouse epithelial cells to ozone (Tan et al., 2018). Similarly, AJC protein and differentiation marker E-cadherin-deficient mice featured several manifestations of asthma, such as progressive epithelial damage, loss of ciliated cells, zones of epithelial denudation, decreased (ZO)-1 expression, and goblet cell metaplasia, globally indicating that the loss of E-cadherin plays a role in the development of asthma epithelial features. Based on the observation that several aeroallergens and viruses have the ability to disrupt E-cadherin-mediated cell–cell adhesion (Post et al., 2018) (see below), one could suggest that asthma epithelial abnormalities may at least partly be driven by aeroallergens-induced E-cadherin disruption. Finally, epithelial basement membrane thickening has also been observed in asthma, particularly involving the reticular lamina. Due to increased collagen deposition in the airway subepithelial area, this feature seems relatively constant all along the trachea–bronchial tree (Jeffery et al., 1989; Wilson and Li, 1997).

Inflammation is a key feature of asthma, and follows the activation of adaptive, type 2 immune responses induced by environmental triggers such as allergens. The AE plays a key role in this inflammatory process by releasing damage-associated molecular patterns (DAMPs) or alarmins, such as TSLP, IL-33, and IL-25, which instruct dendritic cells to polarize T-cell populations into Th2 cells (Edwards et al., 2017).

Finally, basal-to-apical transcytosis is also altered in asthma, as pIgR expression is downregulated in the AE from asthma patients (OrdoÑEz et al., 2000). Concordantly, decreased SC concentrations are found in BALF from asthma patients as compared with controls (Van Vyve et al., 1995). In contrast with COPD, this feature seems independent from the severity of the disease or the allergic background and does not recapitulate *in vitro* upon ALI reconstitution of the AE. The mechanism underlying pIgR downregulation could relate to immune activation through IL-4 receptor (Ladjemi et al., 2018). Interestingly, the “inflammatory memory” that is observed in airway epithelial cells from patients with nasal polyps is thought to be driven through IL-4 receptor activation of basal/progenitor cells (Ordovas-Montanes et al., 2018).

### Cystic Fibrosis

CF is an autosomal recessive disease caused by a mutation in the CF transmembrane conductance regulator (CFTR) gene located in the long arm of human chromosome 7 (Ratjen et al., 2015). The incidence of CF is estimated around 1 in 3,000 live births in Europe but varies according to race and ethnicity (Ratjen et al., 2015). CFTR is an ATP-binding family anion channel whose primary role is to transport chloride and bicarbonate across the apical membrane of epithelial cells. It also regulates (inhibits) the activity of the epithelial sodium channel ENaC, which reabsorbs sodium and water in normal conditions. Among the 2,000 mutations in the CFTR gene that have been described so far, the most frequent is a phenylalanine deletion at position 508 (F508del), leading to a misfolding and destabilization of the CFTR protein, promoting its premature degradation by the proteasome (Ratjen et al., 2015). Consequently, the loss of ENaC inhibition leads to increased sodium influx, resulting in airway surface liquid (ASL) dehydration and decreased periciliary volume, generating in turn cilia compression and affecting muco-ciliary clearance. In addition, the defective CFTR prevents chloride to follow sodium influx, inducing chloride to be retained in the ASL (Krouse, 2001), as the more recently identified alternative chloride channels TMEM16A and SLC26A9 do not appear to counterbalance impaired anion transport in CF patients (Mall and Galietta, 2015; Martin et al., 2018). In addition, decreased bicarbonate secretion associated to an increased H^+^ secretion by ATP12A (an H^+^/K^+^ ATPase channel) in CF patients induces mucus acidification, preventing efficient mucin expansion and generating defective mucus with altered adherence properties (Ratjen et al., 2015). Additionally, ASL acidification seems to impair host defenses and increase the airway bacterial load (Shah et al., 2016). CF lung disease clinically represents a prototypical muco-obstructive disorder, characterized by mucus plugging, chronic neutrophilic inflammation, and recurrent lung infections. This later feature is associated with a worse prognosis, particularly in relation to opportunistic bacteria such as *Pseudomonas aeruginosa* (PA) (Lee et al., 2003).

Decreased mucociliary clearance resulting from viscous mucus stasis is classically observed in CF and participates to increased microbial load and susceptibility to infections (Corcoran et al., 2010). Besides its dehydration and increased viscosity, mucus glycoprotein composition is also altered, with decreased concentrations of MUC5AC and MUC5B as compared with controls, probably due to increased amounts of other mucus components (Henke et al., 2004). Altered cell composition is also found in the CF AE, with hyperplastic goblet and basal cells (Leigh et al., 1995; Burgel et al., 2007).

Although EMT has long been controversial in CF, recent data obtained in CF tissue and cell lines demonstrate EMT occurring in the CF AE. First, CF explants analyzed for EMT markers exhibit increased gene expression for ACTA2, VIM, and COL1A1 and increased protein levels for vimentin and N-cadherin. Similarly, CFBE F508del-CFTR cells cultured in ALI display decreased TEER, along with increased vimentin, N-cadherin, and collagen-1 protein levels (Quaresma et al., 2020). The same study also demonstrates that the presence of (functional) CFTR confers a resistance to TGF-β1-induced EMT, as wt-CFTR cell lines exposed to TGF-β1 show reduced TGF-β1-induced EMT markers as compared with CF mimicking F508del-CFTR cell line. It also revealed that impaired CFTR may lead to EMT in a Twist Family BHLH Transcription Factor 1 (TWIST1)-related manner (Quaresma et al., 2020). In parallel, CF-derived cell lines exhibit increased fibronectin (Nyabam et al., 2016). Transcription factor Kruppel-like factor 4 (KLF4) also seems to play a role in CF AE differentiation, as KLF4-AKT/GSK3β crosstalk, which has been shown to regulate CFTR, is impaired in F508del-CFTR cell lines (Sousa et al., 2020). Of note, CFTR-impaired endothelial cell line also expresses EMT-related signature genes, as shown in a transcriptomic analysis of primary CFTR-impaired and patient-derived endothelial cells. Whereas these signature genes were less expressed than in epithelial cell lines, the use of CFTR modulators reduced the expression of mesenchymal markers (Treps et al., 2021).

Defective apical and gap junctions have also been described in human airway epithelial cells expressing F508del CFTR. Indeed, as compared with ALI cultures of CFTR^+/+^ NuLi cells, CuFi5 CFTR (F508del/F508del) cells exhibited altered TER, along with mistrafficking of connexin 43 (Molina et al., 2015), respectively relating to impaired TJ and gap junctions functionality. In addition, CF_15_ cell line (derived from a F508del/W1282X CF patient) displays impaired protective gap junctions closing upon TNF-α stimulation, which is linked with CFTR dysfunctionality, as adenovirus-mediated wild-type CFTR transfer to CF_15_ cells abrogated this phenomenon (Chanson et al., 2001). Together, these results enlighten both defective physical barrier function and cell–cell communication in CF.

In contrast with asthma or COPD, epithelial polarity-related pIgR expression was unexpectedly upregulated in lung tissue (explants) from patients with end-stage CF (Collin et al., 2020). This finding was in line with increased IgA concentrations in sputum from CF patients but contrasted with the downregulation of pIgR expression upon CFTR dysfunction observed in CF-derived primary cells and CFTR-mutated mice (Collin et al., 2020). Thus, S-IgA immunity seems upregulated in the CF lung despite the genetic imprinting of the epithelium, which is possibly overcome by bacterial-driven mechanisms that include IL-17A production (Collin et al., 2020). This model offers an opportunity of better deciphering the complexity and the respective contribution of inherited versus acquired mechanisms of epithelial barrier dysfunction during such chronic disease. A study comparing wild-type mice with both CF-like CF^MHH^ and CFTR^–/–^ mice also revealed intrinsic alteration of epithelial polarity in CF, witnessed by apical accumulation of β1-integrin in upper airway epithelial cells. This ectopic accumulation of the normally basal integrin arises as a consequence of increased ceramide level. In a vicious circle, ceramide levels are further enhanced by apical β1-integrin-induced acid ceramidase downregulation, leading to lower levels of surface sphingosine and enhanced bacterial infection susceptibility (Grassmé et al., 2017). These results further demonstrate how defective epithelial polarity plays a role in CF physiopathology.

Finally, chronic neutrophilic inflammation is a major feature of the CF AE, and several pro-inflammatory factors (IL-8/CXCL-8, TNF-α, IL-1β, and neutrophil elastase activity) are increased in CF airways as compared with controls (Bergin et al., 2013). In addition, BALF levels in neutrophil granule proteins azurocidin and myeloperoxidase, collected in preschool children with CF, positively correlate with lung damage at 6 years, suggesting that pediatric biomarkers could help predicting the disease progression, strongly linking chronic inflammation with lung destruction (Renwick et al., 2021).

### Idiopathic Pulmonary Fibrosis

IPF is an emerging, fibroproliferative chronic lung disorder, whose increasing incidence currently reaches 9 cases per 100,000 per year (Hutchinson et al., 2015). IPF has a dismal prognosis, with a mean survival of 4 years without treatment (Khor et al., 2020). CS, microaspiration, and viral infections constitute potential triggers of repetitive epithelial injury, leading to alveolar epithelial dysfunction and aberrant wound healing response in genetically susceptible and aged individuals (Chambers and Mercer, 2015). At the histopathological level, IPF classically exhibits usual interstitial pneumonia (UIP) pattern, which encompasses patchy alveolar epithelium injury, increased collagen and other extracellular matrix (ECM) proteins deposition, and varying degrees of fibrotic lesions often resulting in honeycombing (Wilson and Wynn, 2009). Underlying the damaged epithelium, fibroblastic foci constitute one of UIP’s hallmark lesion. In addition, the IPF lung displays a loss of the alveolar epithelium integrity, with disruption of basement membrane and altered TJ (see below). Aside from these fibrotic changes, the IPF lung exhibits abnormal epithelial structures under the form of bronchiolization zones of the alveolar epithelium (colonization of alveolar spaces by migrating bronchiolar cells and/or disorganized local differentiation of alveolar cells), as well areas of hyperplastic alveolar type II cells (AEC2) (Camelo et al., 2014).

AEC2, the progenitor cells of the alveolar epithelium, are thought to be at the root of the disease pathogenesis. Repetitive injury to AEC2 results in exhaustion of their replicative potential, and the secretion of cytokines and growth factors promotes both the recruitment of immune cells and activation of fibroblasts into myofibroblasts (Chapman, 2011; Wynn, 2011). Accumulation of macrophages with a profibrotic phenotype and senescent AEC2 failing to repair alveolar damage ultimately lead to progressive and irreversible lung tissue destruction (Chilosi et al., 2012; Lehmann et al., 2020), orchestrated by epithelial–mesenchymal crosstalk and aberrant reactivation of developmental pathways including WNT/β-catenin, Notch, and Sonic Hedgehog (Carraro et al., 2020; Froidure et al., 2020).

The remodeling of the alveolar epithelium in IPF also results in altered barrier function, with increased epithelial permeability and impaired AJC (Kulkarni et al., 2016). Increased alveolar permeability, witnessed by faster clearance of aerosolized ^99m^Tc-DTPA, was also reported in IPF patients (Mogulkoc et al., 2001). This feature is associated with an altered expression of TJ components by the alveolar epithelium, with increased expression of OCLN, claudin-1, claudin-2, claudin-3, and claudin-7 and downregulation of claudin-18 observed within regions of abnormal epithelialization (Kaarteenaho-Wiik and Soini, 2009; Lappi-Blanco et al., 2013; Zou et al., 2020).

EMT processes have long been thought to play a role in IPF, as persistent type II EMT generates ECM accumulation and tissue remodeling (Kalluri and Neilson, 2003; Kalluri and Weinberg, 2009). However, the role of EMT in IPF, especially in the formation of myofibroblasts, remains controversial following studies that brought scattered results, as reviewed recently (Salton et al., 2019). Nevertheless, alveolar cells adjacent to fibroblastic foci containing α-SMA-positive myofibroblasts (Raghu et al., 2018) may exhibit vimentin expression, putatively reflecting EMT (Yamaguchi et al., 2017).

Aside from their progenitor function, AEC2 lining alveolar spaces also produce surfactant. Surfactant consists of a complex mixture of lipids and proteins whose major function is to exert and maintain low alveolar surface tension, allowing adequate alveolar ventilation and gas exchange (Hamm et al., 1996). While surfactant lipid fraction is mainly constituted by common phospholipids and cholesterol, its protein fraction includes specific surfactant proteins A, B, C, and D (SFTP-A to SFTP-D) (Hamm et al., 1992). Among other roles that are beyond the scope of this manuscript, SFTPs exert innate immune functions allowing viral neutralization, bacteria clearance, and regulation of inflammation (Nayak et al., 2012). IPF patients display pronounced alterations in surfactant properties in BALF (Gunther et al., 1999), while SFTP-A and SFTP-D are increased in serum of IPF patients, possibly due to increased leak from alveolae to interstitium (Greene et al., 2002). While some authors suggest that serum SFTP levels could constitute biomarkers of the disease (Takahashi et al., 2000; Greene et al., 2002), it is also thought that a decrease in serum SFTP-A could reflect outcomes upon antifibrotic drug therapy (Yoshikawa et al., 2020).

## Mechanisms of Epithelial Barrier Dysfunction

**FIGURE 2:**
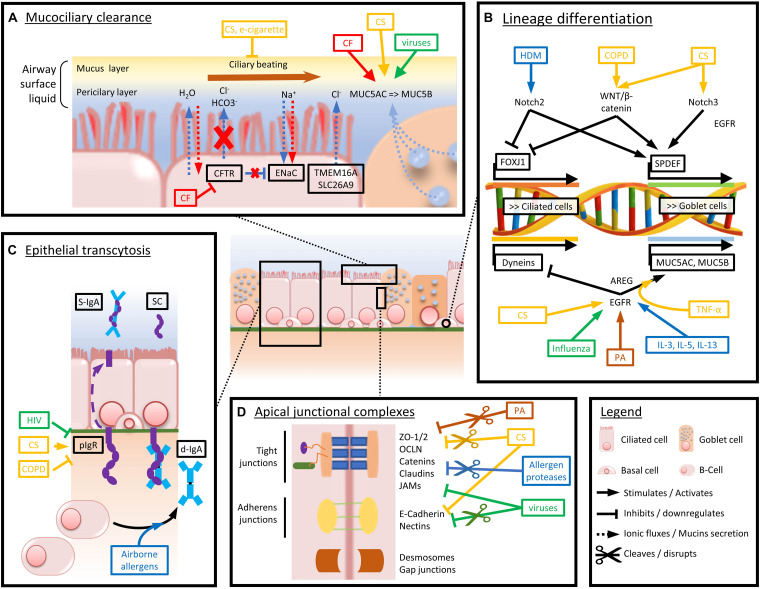
Schematized view of the main mechanisms driving airway epithelium alterations in chronic respiratory diseases. Arrows and boxes are colored depending on the condition or disease driving the represented dysfunctions. Red stands for CF-induced abnormalities, yellow for CS and COPD, green for viruses, brown for bacteria, and blue for airborne allergens and asthma-associated cytokine panels. **(A)** Mechanisms altering mucociliary clearance and mucus composition in chronic respiratory diseases. **(B)** Main factors driving goblet cell hyperplasia and decreased ciliated cell numbers and function. **(C)** Mechanisms driving impaired pIgR expression and d-IgA basoapical transcytosis. **(D)** Mechanisms of altered physical barrier dysfunction (tight and adherens junctions).

A comprehensive view of the major players involved in epithelial barrier dysfunction, extensively described in this chapter, is provided in Figure 2.

### Mucociliary Clearance

Mucociliary clearance in the airways fulfills the removal of inhaled particles from the airway lumen. Coordinated mucus secretion, NaCl and water transport, and cilia beating (Gohy et al., 2016) are different components required for efficient clearance. Goblet cells and submucosal glands secrete mucus, mainly composed of mucins (Ganesan et al., 2013), which are high molecular weight O-linked glycoproteins composed of polymerizing and non-polymerizing forms, and characterized by at least one large region enriched in serine and threonine where glycosylation occurs. Covalent attachment of O-glycans results in expansion of the molecule and confers properties such as protease resistance, pathogen sequestration, and water binding (Thornton et al., 2008). The polymeric mucins MUC5AC and MUC5B are major components of this protective layer and are necessary for efficient gel formation (Thornton et al., 2008). Of note, MUC5AC is primarily produced by goblet cells, while MUC5B is primarily from the submucosal glands (Henke et al., 2004).

Mucins are stored in dehydrated form in secretory granules and secreted upon various physiological or pathological stimuli. MUC5AC secretion seems predominant in normal conditions, while MUC5B is predominant in CF and COPD (Thornton et al., 2008). MUC5AC is upregulated by exposure to viruses, smoke components, and several inflammatory cytokines such as IL-13 (Kuperman et al., 2002; Fahy and Dickey, 2010). Indeed, IL-13 stimulates MUC5AC production through the transcriptional factor activation SPDEF (Takeyama et al., 1999; Fahy and Dickey, 2010), as well as ErbB (such as EGFR) ligands through the activation of MAPK and hypoxia-inducible factor (HIF) pathways. CS also upregulates MUC5AC through EGFR and HIF1α pathways (Yu et al., 2012). Urban PM stimulates MUC5AC production through upregulation of Egr-1, which mediates NF-κB and AP-1 pathways (Xu et al., 2018). According to *in vivo* studies in mice, influenza A upregulates the expression of MUC5AC through EGFR (Barbier et al., 2012). Efficient expansion of the secreted macromolecule depends on several factors such as acidity, calcium concentration, as well as water availability. Under certain circumstances, the secreted mucus is altered, such as in CF where acid dehydrated mucus prevents mucins to expand normally (Thornton et al., 2008).

Periciliary liquid is a less viscous layer that is approximately 7 μm deep, allowing ciliary beating. Its depth is regulated by the amount of liquid and sodium chloride reabsorption, depending on CFTR and ENaC activities (Fahy and Dickey, 2010). While CF displays *per se* impaired anion fluxes, due to altered CFTR functionality, influenza virus is also able to disrupt this anionic transport, through two main distinct mechanisms. First, influenza hemagglutinin binds to the sialic acid residues at the surface of the lung epithelium and downregulates ENaC expression through activation of Src, PLC, and PKC pathways (Kunzelmann et al., 2000; Londino et al., 2017). Second, influenza protein M2 increases lysosomal degradation of CFTR *in vitro* in human kidney cell lines (Londino et al., 2015). The M2 protein also increases proteasomal degradation of ENaC through cellular reactive oxygen species (ROS) increase, either through mitochondrial dysfunction or through activation of the NADPH oxidase (Londino et al., 2017). In addition, parainfluenza virus (Kunzelmann et al., 2004) and respiratory syncytial virus (RSV) (Chen et al., 2009) may alter this anionic transport through similar mechanisms. In addition, external sources of ROS such as air pollution, alcohol, CS, pesticides, and industrial solvents (Phaniendra et al., 2015) have the ability to decrease ENaC activity (DuVall et al., 1998) and to alter periciliary liquid and ciliary beating. Transmembrane mucins also contribute to the physical properties of the periciliary layer. Thus, MUC4 prevents mucus and periciliary layers to be mixed (Fahy and Dickey, 2010), while MUC1 has antimicrobial and anti-inflammatory functions (Fahy and Dickey, 2010), as demonstrated in MUC1-KO mice infected by PA (Umehara et al., 2012; Kato et al., 2016; Chatterjee et al., 2020). *Haemophilus influenzae* also interacts with MUC1, which plays an anti-inflammatory role (Kyo et al., 2012).

Additionally, efficient mucociliary clearance requires a proper ciliary beating in order to enable pathogens and chemicals removal (Fahy and Dickey, 2010). Cilia are located on the airway ciliated cells, representing 50–80% of AE cells (47 ± 2% of airway epithelial cells in the trachea, 73 ± 1% in small airways) (Tilley et al., 2015). In normal conditions, cilia beating frequency is around 12–15 Hz (Salathe, 2007; Fahy and Dickey, 2010). Ciliary motility is provided by dynein ATPases, whose activity is regulated by several proteins that are sensitive to redox environment. Accordingly, ciliary movement is altered upon ROS exposure (Price and Sisson, 2019), including CS. In addition, CS may downregulate dyneins by activating EGFR pathway (Sisson et al., 1994; Tilley et al., 2015). Recently, it appeared that e-cigarette liquids are also harmful for cilia beating, as cinnamaldehyde dysregulates mitochondrial function (Clapp et al., 2019).

Finally, CFTR knockdown in neutrophils reduces spontaneous vanishing of NETs. As NETs promote inflammation by releasing cytotoxic proteases and condensed chromatin, this knockdown favors persistent inflammation and leads to defective resolution of infections (Gray et al., 2018). In addition, the increased amount of DNA from NETotic neutrophils and inflammatory cytokines in the airways further enhance mucus viscosity (Marcos et al., 2015) and mucus plugging (Cheng and Palaniyar, 2013). Infectious agents, mainly PA, *Staphylococcus aureus* (SA) but also viruses, fungi, and protozoa, have been shown to induce NETosis, as well as lipopolysaccharide (LPS) or GM-CSF (Branzk and Papayannopoulos, 2013; Khan et al., 2017).

### AJC and Apico-Basal Polarization

Epithelial AJC promote cell–cell adhesion and barrier integrity and regulate the paracellular passage of ions and macromolecules. AJC comprise both TJ and adherens junctions, while desmosomes and gap junctions provide localized cell–cell adhesion sites, without being part of AJC *stricto sensu*. TJ provide the highest resistance to the epithelium and separate the apical and basolateral poles of epithelial cells and are thereby critical determinants of epithelial polarity (Lamouille et al., 2014). Three types of transmembrane proteins compose TJ: (a) members of the claudin family, (b) MARVEL family members such as OCLN, and (c) Ig-like proteins such as JAMs (Niessen, 2007; Hartsock and Nelson, 2008; Schulzke and Fromm, 2009). E-Cadherin and nectin family members represent the major transmembrane proteins of adherens junctions (Meng and Takeichi, 2009; Indra et al., 2013). E-Cadherin associates with both α- and β-catenins and retains EGFR ligands in adherens junctions, preventing their interaction with the receptor (Qian et al., 2004). Adhesive components of the AJC are stabilized by links to intracellular proteins including ZO proteins, catenin-family proteins, and actin perijunctional belt binding proteins (Ivanov et al., 2010). Besides AJC structures, desmosomes (also known as macula adherens) are localized, spot-like adhesions that allow anchoring to the intermediate filaments of the cytoskeleton (through desmoglein, desmocollin, plakoglobin, plakophilin, and plakin) (Garrod, 2010), while gap junctions are specialized in intercellular communications (Brooke et al., 2012).

Cigarette smoking has long been associated with altered epithelial barrier function and increased epithelial permeability (Hogg, 1982), and the understanding of the underlying mechanisms is steadily growing since. *In vitro*, 16HBE cells and HBEC exposed to CS extract, and smokers-derived ALI-HBEC, display decreased TER (Carlier et al., 2018). Accordingly, downregulation of OCLN, ZO-1, and ZO-2 are observed, along with protein disruption (Heijink et al., 2012; Milara et al., 2013; Schamberger et al., 2014). Similarly, CS-exposed Calu-3 cells show increased tyrosine phosphorylation in OCLN, inducing disruption of OCLN/ZO-1 binding (Olivera et al., 2010). The AE from COPD patients seems even more prone to develop barrier dysfunction as most of junctional genes whose expression is impaired in smokers are further downregulated in COPD (Carlier et al., 2021; Shaykhiev et al., 2011). CS-exposed COPD HBEC also show decreased E-cadherin and ZO-1 expression as compared with HBEC from smokers, possibly due to ROS-dependent decrease in intracellular cyclic adenosine monophosphate (AMP). Interestingly, increased CS effects were not only observed in COPD, as asthma-derived HBEC were more prone than non-smokers to develop barrier dysfunction upon CS exposure (Xiao et al., 2011). Globally, the AE in smokers (and more particularly in COPD patients) displays increased epithelial permeability, probably favoring microbial invasion and COPD exacerbations, which account for a significant part of COPD morbidity (Barnes, 2014; Aghapour et al., 2018; Amatngalim and Hiemstra, 2018).

CS exposure has also been shown to induce EGFR signaling (Shaykhiev and Crystal, 2014), participating in barrier dysfunction. Indeed, EGFR downstream signaling is dysregulated in smokers, leading to squamous metaplasia and EMT, possibly due to CS-related increased amphiregulin (AREG), whose binding to EGFR induces different programming than EGF (Zuo et al., 2016). In addition, CS carries ROS (Zhao and Hopke, 2012), whose multiple effects include enhanced ERK1/2 signaling (Petecchia et al., 2009), decreased EGFR activation (Heijink et al., 2010), decreased EGFR ubiquitination and degradation in bronchial cells (Khan et al., 2008), and hyaluronan fragmentation activating layilin-downstream signaling and RhoA/Rho kinase-dependent E-cadherin decrease (Forteza et al., 2012). *In vitro*, CS-exposed airway epithelial cells display decreased TER, along with junctional components delocalization and downregulation of ZO-1 and OCLN, in an EGFR-dependent manner (Heijink et al., 2010), and E-cadherin disruption secondary to downregulation of A-kinase anchoring protein (AKAP)-9 (Oldenburger et al., 2014). Concordantly, EGF-exposed basal cells display decreased barrier integrity (Shaykhiev et al., 2013). Moreover, CS exposure has been shown to trigger the basolateral migration of MUC1 cytoplasmic tail, which is phosphorylated in an EGFR/Src/Jnk-dependent manner and recruits p120-catenin, further inducing the dissociation of p120-catenin/E-cadherin/β-catenin complex and thus promoting the disruption of adherens junctions (Zhang et al., 2013).

Airborne allergens with proteolytic activity (such as HDM-derived Der p1 allergen) have also been demonstrated to induce epithelial leakiness, facilitating the initiation, and maintain of inflammatory processes driving allergic asthma and allergic rhinitis. In a proof-of-concept study, canine tracheal cells exposed to Der p1 display increased permeability (Herbert et al., 1995), which is at least partly driven by Der p1 direct cysteine and serine proteinase activity on OCLN, claudin-1 (Wan et al., 1999), and to E-cadherin delocalization (Post et al., 2012). Allergenic pollens from numerous plants (including birch, ragweed, cedar, cypress, and several grasses) also release proteases (Gunawan et al., 2008a, b; Takai and Ikeda, 2011), some of which being able to degrade TJ in MDCK cells (Runswick et al., 2007). *Olea europaea*, *Dactylis glomerata*, *Cupressus sempervirens*, and *Pinus sylvestris* allergens also induce variable E-cadherin, OCLN, and claudin-1 disruption in Calu-3 cells ALI-cultures (Vinhas et al., 2011). Cockroach allergen extracts also show complex proteolytic activities, notably through Per a 10 (serine protease) and Bla g 2 (aspartic protease) (Pomes et al., 2002; Sudha et al., 2008), but their action on the epithelial barrier *stricto sensu* is still unclear, although decreased TER in cockroach extract antigen-exposed BEAS-2 and ATCC cells (monolayer cell culture) has been reported (Antony et al., 2002; Lee et al., 2018). Besides its hypothetical effects on barrier permeability, Per a 10 induces IL-6, IL-8/CXCL-8, and GM-CSF in A549 cells (Kale and Arora, 2015). Finally, several food allergens with proteolytic activity affect intestinal epithelial barrier integrity, as reviewed in Gavrovic-Jankulovic and Willemsen (2015), but the description of the underlying mechanisms is beyond the scope of this review.

In two similar studies, RV-exposed 16HBE14O^–^ ALI cultures display decreased TER and increased bacteria (Sajjan et al., 2008) and allergen penetration (Gangl et al., 2015). In exposed mice and 16HBE14o^–^ cells, RV also leads to decreased junctional ZO-1 expression (Sajjan et al., 2008). Mechanistically, RV interacts with Nod-like receptor (NLR) Family Member X1 (NLRX1), a double-strand (ds) RNA receptor, and drives mitochondrial ROS generation that will impair the epithelial barrier (Unger et al., 2014). Differently, in colonic Caco-2 cell line, group B coxsackie viruses attach to glyosyl-phosphatydilinositol-anchored protein decay-accelerating factor, therefore inducing Abl and Fyn kinase signals and allowing the virus to bind to the normally unreachable coxsackievirus adenovirus receptor, entering the cells in an OCLN-dependent manner (Coyne et al., 2007), ultimately leading to decreased TER and TJ disassembly (Coyne and Bergelson, 2006). Influenza virus has also been shown to induce alveolar barrier disruption in NCl-H441 cell line, with decreases in TER, JAMs, and claudin-4 expression (Short et al., 2016). In porcine bronchial cells cultured in ALI, however, influenza does not alter TER but leads to loss of ciliated cells and thinner epithelium layer (Wu et al., 2016). Similarly, respiratory syncytial virus (RSV) infection drives barrier dysfunction by decreasing the expression of TJ proteins (claudin-1, OCLN, ZO-1), disrupting E-cadherin in murine and human bronchial cells (Smallcombe et al., 2019) and inducing cytoskeletal remodeling in ALI 16HBE14o^–^ cells (Rezaee et al., 2013). Contrary to the gut epithelium, where lay abundant commensal bacterial populations constituting the gastrointestinal microbiota, the recently described airway microbiota is thinner and less well defined (O’Dwyer et al., 2016). Therefore, bacteria/epithelium interactions have been more widely studied in the gut, as reviewed by Barreau and colleagues (Barreau and Hugot, 2014), but the highlighted mechanisms are not transposable at the respiratory level due to different microbiota compositions. Nevertheless, PA, common respiratory pathogen in immunocompromised patients (more particularly in COPD and CF) (Murphy, 2009), induces barrier dysfunction in the lung in many ways, as recently reviewed (Ruffin and Brochiero, 2019). First, galactophilic internal lectin of PA has a direct dose-related toxicity toward respiratory epithelial cells (Bajolet-Laudinat et al., 1994). Second, PA produces proteases that disrupt TJ and contribute to tissue damage in respiratory infections (Hobden, 2002; Kipnis et al., 2006). Third, PA’s quorum sensing 3-oxo-C12-HSL is able to interfere with mitochondrial biogenesis and induce TER decrease in BEAS-2B cells (Maurice et al., 2019) but not in HNCN cells cultured in ALI, suggesting that prior epithelial damage is needed for PA to exert its deleterious effects (Losa et al., 2015). Several studies also report SA-induced barrier dysfunction in the upper AE. Human nasal epithelial cells cultured in ALI exposed to secreted products from SA (Malik et al., 2015) or purified SA V8-protease (Murphy et al., 2018) display decreased TER and disrupted TJ and decreased TER and delocalized ZO-1, respectively. Interestingly, nasal mucosa from healthy donors did not display barrier dysfunction when exposed to SA, in contrast with the nasal mucosa from patients with chronic rhinosinusitis with nasal polyps, suggesting that a “pre-lesion state” conditions this effect on the epithelial barrier (Altunbulakli et al., 2018). Interestingly, aberrant intracellular signaling in CF cells may cause an ectopic expression of fibronectin/β1-integrin at the apical side of epithelial cells, providing docking sites for PA (Grassmé et al., 2017). Finally, diverse fungi can alter the barrier function. Pen ch 13, a serine protease allergen issued from airborne fungi such as *Aspergillus* and *Penicillium*, is able to cleave OCLN in 16HBE14o^–^ cells (Tai et al., 2006), while 16HBE14o^–^ cells and ALI-HBEC from asthmatic donors exposed to *Alternaria alternata* extracts show decreased TER (Leino et al., 2013). In addition, fungal hyphae secrete proteases and toxins that impair epithelial integrity, possibly by increasing IL-6 and IL-8/CXCL-8 release, as shown in A549 cells (Tomee et al., 1997; Kauffman et al., 2000; Chaudhary and Marr, 2011).

Ambient PM and one of its subsets, diesel exhaust, constitute an important risk factor for developing or worsening chronic respiratory diseases (Wu et al., 2018; Zhao et al., 2019). It does notably participate to inducing airway barrier dysfunction. HNEC exposed to PM2.5 and 16HBE human bronchial cells exposed to outdoor PM both displayed decreased TER and TJ protein expression (Rezaee et al., 2010; Xian et al., 2020). PM also generates ROS production (see above) and inflammation (Baulig et al., 2003; Cooper and Loxham, 2019), further inducing epithelial leakiness and increased susceptibility to invading pathogens, as demonstrated in PA-infected mice exposed to PM (Liu et al., 2019).

### Epithelial Transcytosis

Although second in serum behind IgG, IgA is the predominant Ig at mucosal surfaces and acts as a crucial actor in mucosal immunity (Pilette et al., 2001a). IgA possesses multifaceted functions that encompass immune exclusion, neutralization of antigens and pathogens plugged in mucus, regulation of microbiota, as well as regulation of immune cells in the submucosa (Carlier et al., 2016). At mucosal sites, IgA is produced by dedicated plasma B cells in its dimeric form (d-IgA). D-IgA is then transported across the epithelium by the pIgR to reach mucosal secretions where it is released as secretory (S)-IgA (see above) (Pilette et al., 2001a). Two subclasses of IgA coexist in humans, with IgA_1_ predominating in serum as monomers, while IgA_2_ is enriched in external secretions (mainly as dimers), representing up to 50% of total IgA (Delacroix et al., 1982). To produce IgA, naive B cells mature through IgA class switching. Class switch recombination toward IgA depends on multiple mediators such as TGF-β, B-cell activation factor (BAFF), A proliferation-inducing ligand (APRIL), TSLP, and IL-6 (Joo et al., 2017), as well as by external factors such as microbial stimuli acting on lung dendritic cells, through MyD88-dependent Toll-like receptor (TLR) activation (Ruane et al., 2016).

The effect of CS exposure on pIgR expression remains unclear. Thus, CS seems to stimulate the gene expression of pIgR *in vivo*, possibly due to increased inflammatory factors TNF-α and IFN-ɣ, but this is observed at the transcriptional level only, suggesting that CS exposure might also induce posttranscriptional modifications in pIgR protein synthesis (Gohy et al., 2014). In addition, *in vitro* studies on HBEC revealed that CS rather induces a decrease in pIgR that is linked to a dedifferentiation of the AE (Rusznak et al., 2001; Amatngalim et al., 2018). CS-related pIgR downregulation is further aggravated in COPD, as ALI-HBEC from COPD patients displayed decreased pIgR protein expression as compared with smokers without COPD (Carlier et al., 2021). This alteration is at least partly due to COPD-related increased TGF-β signaling (Gohy et al., 2014).

The pIgR/IgA system may also be disrupted by microorganisms. According to *in vivo* studies on macaques, pIgR is downregulated in HIV-infected macaques, in an IL-17-mediated manner (Jaffar et al., 2009; Li et al., 2017). This mechanism could be involved in recurrent opportunistic respiratory infections presented by HIV-patients. A study comparing wild-type and pIgR^–/–^ mice bred in normal versus germ-free housing conditions illustrated the relationship between pIgR/IgA system and microbiota, as pIgR-deficient mice developed upon aging a COPD-like phenotype in normal housing conditions, including increased numbers of neutrophils and macrophages in their BALF, emphysema, and peribronchiolar inflammation. In contrast, wild-type mice (in both conditions) and pIgR^–/–^ mice bred in germ-free environment showed normal BALF counts and lung histology, underscoring the importance of pIgR/IgA in lung mucosal defense (Richmond et al., 2016).

Of note, lower S-IgA concentrations were also found in BALF from lung transplant recipients with acute rejection as compared with controls (Bastian et al., 2000), whereas allergen challenge in patients with allergic asthma or allergic rhinitis increased BAFF concentration in BALF, potentially favoring IgA class switch recombination in these patients (Kato et al., 2009).

### Cell Composition of the Epithelium: Lineage Differentiation and Repair

Adequate differentiation of the AE provides condign cell populations and optimal epithelial structure and functionality. In chronic respiratory diseases, however, both lineage differentiation and repair capacity may be altered, as described previously in this manuscript.

CS is classically associated to goblet cells hyperplasia and decreased ciliated cells numbers (Saetta et al., 2000; Kim et al., 2015). Even though numerous studies have been led in the field, the precise mechanisms of these effects are not yet fully elucidated. In ALI-HBEC, exposure to CS component acrolein (but not formaldehyde and acetaldehyde) induces increased percentage of MUC5AC^+^ cells (Haswell et al., 2010), while total CS extract increases goblet and club cells numbers while reducing the number of ciliated cells (Schamberger et al., 2015). Similarly, in a recent study, HBEC from healthy non-smokers displayed increased numbers of goblet MUC5AC^+^ and decreased numbers of club SCGB1A1^+^ cells upon CS exposure (Gindele et al., 2020). Mechanistically, these changes were shown to depend on Notch-3 activation, highlighted by nuclear migration of the Notch3 intracellular domain (NICD3). In addition, NICD3-treated HBEC exhibited sharp increases in SPDEF and MUC5AC, corroborating Notch-3-induced enhanced differentiation toward goblet cells (Bodas et al., 2021). *In vivo* studies on mice further show that HDM exposure activates Jag1/Notch2 signaling, more particularly in small airways alternative progenitors (club cell)s. Jag1/Notch2 activation subsequently induces both downregulation of ciliated cells marker FOXJ1 and upregulation of goblet cells transcriptional factor SPDEF (Carrer et al., 2020), mimicking two central features of the asthma epithelial phenotype and further suggesting a role for Jag1/Notch2 signaling in the pathogenesis of abnormal epithelial lineage in asthma. In parallel with these results, IL-13-exposed ALI-HBEC display increased percentage of MUC5AC^+^ cells (Haswell et al., 2010). Aside from Notch abnormal reactivation, aberrant WNT/β-catenin reactivation also contributes to impaired epithelial cell differentiation reminiscent of COPD, as extrinsic activation of WNT/β-catenin in ALI-HBEC increased SPDEF gene expression while suppressing expression of ciliated cells-associated genes FOXJ1 and MCIDAS (Malleske et al., 2018; Carlier et al., 2020).

In addition to developmental pathways, EGFR signaling pathway is widely reported to modify cell populations in the AE. More than 20 years ago, Takeyama and colleagues reported in a proof-of-concept study that EGFR ligands promote the expression of MUC5AC, an effect that is potentiated by TNF-α, while EGFR signaling inhibition blocks differentiation toward goblet cells (Takeyama et al., 1999). In addition, while ovalbumin-induced asthma and inhaled TNF-α-induced airway inflammation in rats lead to increased EGFR expression and goblet cell production in the AE, these effects are abrogated by prior treatment with EGFR inhibitor BIBX1522 (Takeyama et al., 1999). Since then, the complexity of EGFR downstream pathways has been unraveled, as well as its many ligands, as reviewed elsewhere (Vallath et al., 2014). For example, CS-induced EGFR ligand amphiregulin (AREG), whose expression is induced by CS, drives a specific EGFR activation pattern in basal cells that differs from EGF-driven squamous metaplasia. Indeed, AREG-related EGFR downstream signaling induces basal and goblet cell hyperplasia, along with impaired ciliated cell differentiation and repressed EGF expression (Zuo et al., 2016). Impaired EGFR signaling has also been linked with excessive goblet cell differentiation in asthma (Takeyama et al., 2001) and increased mucin expression in CF (Burgel et al., 2007). Strikingly, NCI-H292 airway cell line exposed to PA showed activated EGFR and increased MUC5AC expression, linking opportunistic bacterial invasion with EGFR signaling pathway and increased mucus secretion (Kohri et al., 2002). Finally, asthma-related Th2 cytokines such as IL-4, IL-5, and IL-13 can induce EGFR expression (Wills-Karp, 2004), while cytokines produced by activated eosinophils such as IL-3 and IL-5 can induce TGF-α production by epithelial cells and subsequently induce mucins production through EGFR signaling (Burgel et al., 2001).

### Innate Immunity

Besides its physical barrier and mechanical removal of inhaled pathogens by mucociliary clearance, the AE possesses other defense mechanisms, including the recognition of microorganisms, the secretion of chemo/cytokines, or the active production of antimicrobial substances (Parker and Prince, 2011; Hiemstra et al., 2015).

#### Host Defense Peptides

Host defense peptides of the airways (also known as antimicrobial peptides) include β-defensins, cathelicidins, lysozyme, lactoferrin, secretory leukocyte protease inhibitor (SLPI), and elafin, all of them targeting bacteria, viruses, parasites, and/or fungi by diverse mechanisms.

β-Defensins are small cationic peptides that interact with microbial lipids and cover Gram-positive and Gram-negative bacteria as well as enveloped viruses and fungi (Sorensen et al., 2008). Among their numerous other roles (Meade and O’Farrelly, 2018), β-defensins may attract immune cells and activate dendritic cells (Yang et al., 1999). Polymorphisms in the β-defensin-1 gene have been related to asthma and COPD susceptibility (Matsushita et al., 2002; Levy et al., 2005). In ALI-HBEC, exposure to PA induced physiological β-defensin-2 release, which was abrogated by CS exposure. In addition, β-defensin-2 levels in pharyngeal washes from patients with community-acquired pneumonia were lower in both current and former smokers, as compared with never smokers (Herr et al., 2009). Conversely to these findings, β-defensins 3, 5, and 9 are upregulated in CS-exposed A549 cells (Pierson et al., 2013), while increased β-defensin-1 levels were found in sputum supernatants from COPD and severe asthma patients. Additionally, β-defensin-1 are decreased in supernatants from CS-exposed control HBEC but not COPD-derived HBEC (Baines et al., 2015). These apparently discordant results were also found in oral Leuk-1 cells where CS-exposure reduced β-defensin-1 while enhancing β-defensin-2 and β-defensin-3 levels (Wang et al., 2015). In addition, A549 cells exposed to PM exhibit dysregulated expression of β-defensin-2 (Rivas-Santiago et al., 2015), while BEAS-B2 cells infected with PA showed altered β-defensin-2 when primed with PM (Chen et al., 2018).

Cathelicidins regroup a family of three highly cationic peptides (including the most studied LL-37) that interact with negatively charged bacterial membranes and have antimicrobial effects against fungi, parasites, and viruses. They are constitutively secreted in low concentrations by epithelial cells, but higher local concentrations may be reached by neutrophil degranulation (van Harten et al., 2018). In sputum from COPD patients, increased LL-37 levels were observed, as well as in CS- and LPS-exposed respiratory cells (BEP2D and A549 cell lines) (Jiang et al., 2012). In contrast, another study showed that LL-37 supplementation restores CS-induced disruption of OCLN and ZO-1 in ALI-cultured Calu-3 cells (Tatsuta et al., 2019). LL-37 is also able to induce eosinophil cationic protein and cysteinyl leukotrienes release in eosinophils, which was further enhanced both in cells from asthmatic patients and in GM-CSF- or IL-5-primed cells (Sun et al., 2013). Similarly, LL-37-exposed cocultures of eosinophils and BEAS-2B cells displayed increased IL-6, IL-8/CXCL-8, and CCL4, while murine LL-37 homolog cRAMP increased bronchial hyperresponsiveness in ovalbumin-induced murine asthma (Jiao et al., 2017). Finally, although no study has yet explored whether PM could alter cathelicidins, carbon nanoparticles inhibit LL-37 antibacterial and antiviral properties *in vitro* (Findlay et al., 2017).

Lysozyme is one of the most abundant protein in human airway secretions. Secreted by submucosal glands, neutrophils, and macrophages, it has dual activities as lytic enzyme and cationic protein, targeting peptidoglycans from bacterial walls and disrupting their membranes (Ragland and Criss, 2017). Lysozyme levels were unchanged in CS-exposed mice (Shibata et al., 2008), but smokers and e-cigarette users, respectively, exhibited increased and decreased sputum lysozyme levels as compared with non-smokers (Reidel et al., 2018). Similarly, an older study showed increased lysozyme secretion from smokers’ alveolar macrophages when compared with non-smokers (Hinman et al., 1980).

Finally, lactoferrin, an iron-binding glycoprotein mainly produced by neutrophils, which damages the outer membrane of Gram-negative bacteria (Caccavo et al., 2002), has been found to control bacterial growth in the airways, while its function is impaired in BALF from smokers (Vargas Buonfiglio et al., 2018). SLPI, a low-molecular weight antiprotease that is produced by goblet cells (Doumas et al., 2005), is increased in CS-exposed mice (Shibata et al., 2008) and in nasal epithelial cells from smokers in a STAT1-dependent manner (Meyer et al., 2014). However, it may be inactivated by oxidative stress, impairing its antimicrobial functions (Taggart et al., 2001). Following RV infection, SLPI levels in sputum from COPD patients were significantly decreased as compared with (non-)smokers controls, precipitating secondary bacterial infections (Mallia et al., 2012). SLPI also inhibits airway hyperresponsiveness in ovalbumin-sensitized mice and guinea pigs and in ascaris-sensitized sheep (Wright et al., 1999; Nandedkar et al., 2004). In addition, SPLI mRNA expression from human bronchial brushes are decreased in severe asthma as compared with mild/moderate asthma patients (Raundhal et al., 2015).

#### Toll-Like Receptors

Pathogen-associated molecular patterns (PAMPs) are pathogen-specific, invariant molecular structures that can be bound by pattern recognition receptors (PRR), therefore contributing to microbial detection by airway epithelial cells, and to initiation of immune response. PRR are divided into four families, including TLRs that are expressed by most airway epithelial cells. After binding PAMP, PRR initiate downstream signaling (for instance through MyD88 and TRIF in the case of TLR), ultimately leading to immune response (Leiva-Juarez et al., 2018). In respiratory diseases, TLR2 and TLR4 are most relevant, as described hereunder. TLR2 plays a role in detecting Gram-positive bacteria, while TLR4 recognizes Gram-negative bacteria through LPS (Jiang et al., 2006; Lafferty et al., 2010).

When exposed to the viral PAMP poly(I:C), mice display increased parenchymal and bronchiolar inflammation, remodeling, and apoptotic responses, which are sharply increased upon prior CS exposition (Kang et al., 2008). TLR3-dependent and TLR3-independent signaling, along with IFN-γ- and PKR-dependent mechanisms, were shown to participate to this CS/poly(I:C) interaction (Kang et al., 2008). More recently, CS and viral PAMP synergistic mechanisms to induce airway inflammation were further documented, as mice exposed to CS plus poly(I:C) or influenza A displayed increased inflammation and remodeling, which resulted from 2′-5′oligoadenylate synthetase/RNase L activation (Zhou et al., 2013). Besides its deleterious synergy with viral PAMP, CS alone also induces TLR4 expression, *in situ* and *in vitro* in 16HBE cells (Pace et al., 2008; Geraghty et al., 2011). CS-related TLR4 signaling was shown to increase MMP-1, a crucial actor in emphysema, in a MyD88- and IRAK1-dependent manner (Geraghty et al., 2011), while CS-exposed TLR4^–/–^ mice were protected from emphysema, impaired lung function, airway fibrosis, and collagen deposition in small airways as compared with wild-type mice. On the contrary, these features were increased in CS-exposed TLR2^–/–^ mice, suggesting a protective role of TLR2 in the lung (Haw et al., 2018).

Lung cells expressing TLR4 recognize LPS contaminating HDM, and TLR-4 signaling participates in HDM sensitization through LPS-induced IL-1α release, which in return targets epithelial cells in an autocrine manner (Willart et al., 2012). This leads epithelial cells to produce TSLP, GM-CSF, IL-25, and IL-33, ultimately driving T-lymphocytes toward a Th2 phenotype (Hammad et al., 2009). The role of TLR4 in promoting asthma was further explored by inhibiting or silencing the gene. Thus, HDM-exposed mice (both wild-type mice that inhaled TLR4 inhibitor and TLR4^–/–^ mice) were shown to display reduced asthma features (Smits et al., 2009). In addition, polymorphisms of the TLR4 gene protect bakery workers from developing work-related respiratory symptoms (Cho et al., 2011), and SNPs on the same gene are thought to influence asthma severity (Zhang et al., 2011). Similarly, polymorphisms in the TLR2 gene were also associated with modified risk of developing asthma (Qian et al., 2010; Gao et al., 2013).

Although TLR profile expression comparison in control versus CF epithelial cell lines (16HBE14o and CFTE29o, respectively) and in wild-type versus CFTR^–/–^ mice globally brings similar patterns (Muir et al., 2004; Greene et al., 2005), PA-exposed CF cells (IB3-1) produce more IL-6 than “CF-corrected” cells (C38), in a manner depending from TLR5/bacterial flagellin interaction (Blohmke et al., 2008). Moreover, CF patients display increased TLR5 levels in airway neutrophils than controls and patients with non-CF bronchiectasis (Koller et al., 2008), while TLR5^–/–^ mice exposed to flagellated PA displayed reduced neutrophil recruitment as compared with both wild-type mice and TLR5^–/–^ mice challenged with non-flagellated PA (Morris et al., 2009), further underlining the specific contribution of TLR5/flagellin interaction to inflammation in CF. Interestingly, not only microorganisms can drive TLR signaling in the CF lung, but also microbleeds in the CF lung may result in the presence of heme, which can activate TLR signaling and inflammatory cytokine release in CFBE41o– CF cell line (Cosgrove et al., 2011), while increased neutrophil elastase is able to directly activate TLR4, ultimately leading to increased IL-8/CXCL-8 production (Devaney et al., 2003).

#### DAMP/Alarmins

Aside from external triggers driving inflammation (for instance, through PAMP), epithelial injury also results in the release of danger signals, called DAMP or alarmins, although some authors subtly differentiate those terms (Bianchi, 2007; Kaczmarek et al., 2013). DAMP trigger the activation of inflammatory pathways independently from microbial invasion, leading to “sterile inflammation” (Zindel and Kubes, 2020). A large range of protein can act as DAMP upon cell damage, a function mediated by their mislocalization or altered structure. By inducing a switch from apoptosis toward necroptosis in neutrophils, CS induces the release of high motility group box 1 (HMGB1) *in vitro*, which further leads to increased IL-8/CXCL-8 secretion by epithelial cells (Heijink et al., 2015). Similarly, CS exposure of BEAS-2B cells and mice leads to increased necroptosis and to HMGB1 and heat shock protein-70 (HSP70) in culture supernatants and BALF, respectively (Pouwels et al., 2016). *In vivo*, increased levels of HMGB1 were observed in BALF from smokers with COPD as compared with healthy (non-COPD) smokers (Ferhani et al., 2010), while increased serum levels of HSP70 and HSP27 are even proposed as diagnostic markers for COPD (Hacker et al., 2009). Not only CS can induce DAMP release, as ovalbumin-sensitized mice display increased HMGB1 expression, but also the addition of exogenous HMGB1 increases Th2 cells and asthma-related cytokines such as IL-4 and IL-17 (Ma et al., 2015). In humans, increased sputum levels of HMBG1 were observed in asthmatics’ sputum (Hou et al., 2011; Watanabe et al., 2011), corroborating these findings. IL-1α, another DAMP, is also thought to play a role in asthma and COPD. Thus, IL1R^–/–^ mice display reduced capacity to mount Th2 responses to HDM (Willart et al., 2012), while CS-exposed cocultured fibroblasts and bronchial epithelial cells from COPD patients show increased inflammatory response, driven by a stronger IL-1α response than control cocultures (Osei et al., 2016).

### The Respiratory Epithelium as Regulator of Immune Homeostasis

Besides its physical barrier and innate immune functions, the AE is able to sense alterations of the luminal contents and to drive adapted immunity through resident and recruited immune cells, as recently reviewed (Hewitt and Lloyd, 2021). Macrophages are the prominent immune cells in the airway lumen, and their interaction with epithelial cells ensures immune homeostasis, namely, an adequate balance between immune tolerance to innocuous airborne pathogens and effective defense against external insults (Puttur et al., 2019). As an example, airway macrophages physically interact with epithelial cells through connexin-43-containing GAP junctions in order to restrict inflammatory response to LPS (Westphalen et al., 2014). In addition, BALF from allergic asthma patients who were challenged with airborne allergens contain increased levels of colony-stimulating factor 1 (CSF1). CSF1 is secreted by the AE and was further shown to promote airway inflammation, as mice with CSF1 deletion restricted to the AE exhibited reduced airway inflammation. CSF1 also increases the numbers of a subset of dendritic cells, highlighting interactions between epithelial cells and dendritic cells (Moon et al., 2018). The AE also participates to replenishing CD8^+^ tissue-resident memory T cells (T_RM_) of the airways and interstitium by constitutively secreting CXCL-16 (Takamura et al., 2019). In addition, a subset of these T cells expresses CD103, allowing them to interact directly with epithelial cells by binding to E-cadherin, therefore promoting their local retention (Higgins et al., 1998). Another T_RM_ population (CD4^+^ T_RM_) is increased in pneumococcal pneumonia and regulates lung epithelial cells to produce CXCL-5, enhancing neutrophil recruitment and optimizing mucosal defense (Shenoy et al., 2020). Resident immune cells are also able to impede epithelial cell response to pathogens. Thus, IFN-λ decreases airway regeneration in a murine model of influenza infection (Major et al., 2020), while poly(I:C)-challenged mice leads to IFN-λ production by dendritic cells, impairing airway barrier and increasing susceptibility to bacterial superinfections (Broggi et al., 2020).

## Epithelium–Mesenchyme Crosstalk in Respiratory Diseases

Epithelial–mesenchymal crosstalk orchestrates lung development and normal wound healing processes (Evans et al., 1999; Thiery et al., 2009). The so-called “epithelial–mesenchymal trophic unit” (EMTU) has been proposed as structural and functional element in these processes (Evans et al., 1999). In lung organogenesis, EMTU is composed of an attenuated fibroblast sheath formed by interweaved (myo)fibroblasts, attached to the reticular basement membrane, which supports the lung epithelium (Osei and Hackett, 2020). More than 20 years ago, Evans and colleagues suggested that the lung EMTU is reactivated in lung diseases, driving aberrant repair responses (Evans et al., 1999), and recent advances in 3D *in vitro* coculture systems have unlocked new tools to study epithelial–mesenchymal interactions in health and disease, allowing to revisit this statement.

In COPD, CS exposure damages the AE, as thoroughly described previously, inducing the release of DAMP (e.g., IL-1α, HMGB1), proinflammatory cytokines (IL-8/CXCL-8, IL-6, TNF-α), antimicrobial peptides such as LL-37, as well as profibrotic growth factors (TGF-β) (Pouwels et al., 2016; Di Stefano et al., 2018). These mediators, along with recruited immune cells, activate underlying fibroblasts to increased ECM production, leading to airway remodeling (Annoni et al., 2012; Brandsma et al., 2020). Mechanistically, it has been shown that damaged airway epithelial cells trigger fibroblasts to release IL-6, IL-8/CXCL-8, and GM-CSF (Suwara et al., 2014). As DAMP, IL-1α may be released following varied epithelial pathways, such as following endoplasmic reticulum stress activation (by thapsigargin) (Suwara et al., 2014), viral infection [either by viral mimic poly(I:C) or directly by RV exposure] (Hill et al., 2016), or CS exposure (Pauwels et al., 2011). When exposed to IL-1α, lung fibroblasts release increased amounts of IL-6 and IL-8/CXCL-8, along with HSP-70 (Osei et al., 2016). Inhibiting the IL-1α axis with neutralizing antibodies represses this inflammatory response (Suwara et al., 2014), while IL-1 pathway inhibitor miR-146-5p is downregulated in fibroblast–epithelium co-cultures from COPD patients as compared with controls (Osei et al., 2017). Collectively, these results point to IL-1α signaling as a key molecular switch of epithelial–mesenchymal crosstalk, with increased signaling in COPD that reactivates EMTU.

Apart from contributing to inflammatory changes, epithelial–mesenchymal crosstalk also participates to fibrosis and EMT in COPD. In airway cells/fibroblast cocultures, CS-exposed airway cells release DAMP LL37, inducing collagen production by fibroblasts. This was not obtained upon direct fibroblast exposition to CS, confirming impaired epithelial–fibroblast interaction (Sun et al., 2014). In addition, changes in gene and protein expression are described in the COPD AE undergoing squamous metaplasia. Thus, increased IL-1α and IL-1β gene and protein expression have been reported in these zones. IL-1β has then been shown to trigger profibrotic responses in surrounding fibroblasts, which rely on integrin-mediated TGF-β activation (Araya et al., 2007). In contrast, airway epithelial cells exposed to fibroblast-conditioned culture medium exhibit increased EMT markers and transcription factors (Nishioka et al., 2015), demonstrating the bidirectional nature of epithelial–mesenchymal crosstalk.

In asthma, ALI-cultured controls airway epithelial cells were exposed to RV and cocultured with airway smooth muscle cells (ASMC) from either controls or asthmatic patients. Asthma-derived, but not control-derived, ASMC released a series of inflammatory chemo/cytokines, among which CCL-5 increases monocyte migration (Allard et al., 2019). In line with data reporting increased IL-1α in BALF from asthma patients (Willart et al., 2012), airway epithelial cells from asthma patients released more IL-1α that controls in early redifferentiation time points in ALI culture, and IL-1α induced proinflammatory responses in fibroblasts (Osei et al., 2020), further supporting the role of IL-1α in dysregulating of EMTU in chronic respiratory diseases. Finally, BEAS-2B cells treated with brain natriuretic peptide were shown to release acetylcholine that, in turn, induces increased expression of inducible nitric oxide synthase and myosin phosphatase target subunit-1 in asthmatic-derived ASMC as compared with control ASMC. This further leads to decreased histamine sensitivity, and asthmatic (but not control) ASMC reduced hypercontractility, further complexifying the interactions into the EMTU in asthma (Calzetta et al., 2014).

Likewise, IPF epithelial–mesenchymal crosstalk has been increasingly studied in the last years, based on the observation that alveolar cells adjoining fibroblastic foci exhibit salient phenotypic changes (Horowitz and Thannickal, 2006). Upon repetitive microinjuries, activated AEC2 notably exhibit reactivation of developmental pathways (WNT/β-catenin, SHH) and secrete fibrogenic growth factors and cytokines (PDGF, TGF-β) (Stewart et al., 2003; Konigshoff et al., 2009), leading to fibroblast activation into myofibroblasts that deposit increased amounts of extracellular matrix (Hung et al., 2013). In turn, impaired fibroblast biology modifies the phenotype of surrounding AEC2, reactivating developmental EMTU. For instance, AEC2 exposed to IPF fibroblast-conditioned media lead to aberrant epithelialization, a surrogate of IPF-related bronchiolization. In this study, further secretome analysis identified SPARC as key paracrine mediator of fibroblast-induced epithelial activation (Conforti et al., 2020). In parallel, bronchiolar smooth muscle cell progenitors in the distal mesenchyme produce WNT-dependent FGF10, allowing distal lung to repair after injury via differentiation of bronchial stem cells (Volckaert et al., 2011). In murine bleomycin-induced lung fibrosis, inactivation of the FGF10/FGFR2B axis led to increased honeycombing lesions, while FGF10 overexpression enhanced fibrosis resolution (Yuan et al., 2019), further deciphering the complex network of signaling pathways that operate in IPF.

## Targeting the Airway Epithelium in Chronic Respiratory Diseases

Inhaled medications are the cornerstone of the treatment of asthma and COPD, with bronchodilators (β_2_-agonists and antimuscarinics) and inhaled corticosteroids (ICS) providing various degrees of improvements in lung function, exacerbations, and symptoms. Inhaled therapies allow higher drug deposition into the airways than oral/systemic treatments and offer the advantage of bypassing to a large extent the related systemic adverse events (Virchow et al., 2008). As first barrier encountered by airborne particles, the AE is exposed to high doses of inhaled pharmacological therapies, and their effect on barrier function has been explored, as briefly reviewed below. Inhaled treatment is less-well defined in CF, while both bronchodilators and ICS may be used along with inhaled DNAse in order to fluidify mucus and inhaled antibiotics to treat or prevent bacterial infections (Agent and Parrott, 2015). In IPF, current inhaled therapies are facing the difficulty to reach the distal and alveolar areas. In addition, only systemic antifibrotic drugs (nintedanib, pirfenidone) have been shown to be effective on the course of the disease, whereas their impact on the epithelial barrier function remains unknown.

Evidence shows that ICS may enhance airway epithelial barrier integrity. Calu-3 and 16HBE cells treated with dexamethasone, fluticasone propionate, or budesonide display increased TER, with dexamethasone-ameliorating TJ assembly (Sekiyama et al., 2012). Similarly, ALI cultures from NCIH441 cells and primary tracheal epithelial cells exposed to dexamethasone (but not hydrocortisone) display decreased paracellular permeability, higher TER, and increased claudin-8 expression, a protein that is required to recruit OCLN at the TJ (Kielgast et al., 2016). In addition, ICS improve barrier function *in vitro* and *in vivo* in a murine asthma model (Steelant et al., 2016). Besides their effects on barrier function, glucocorticoids also act on goblet cell hyperplasia, as ovalbumin-sensitized mice receiving dexamethasone were protected from developing goblet cell hyperplasia upon allergen challenge, while challenged ovalbumin-sensitized mice showed accelerated resolution of this feature when treated with dexamethasone (Blyth et al., 1998). Dexamethasone also inhibits MUC5AC expression in primary epithelial cells and A549 and NCI-H292 cell lines, although with conflicting results (Lu et al., 2005; Chen et al., 2006). *In vivo*, daily treatment with budesonide did not modify MUC5AC and MUC5B expression in bronchial biopsies from patients with mild asthma (Groneberg et al., 2002). Finally, ICS treatment results in diminished airway inflammation, as extensively reviewed and beyond the scope of this review. Briefly, at the epithelial level, a large meta-analysis demonstrates that ICS treatment decreases fractional exhaled nitric oxide (FeNO) levels in asthma patients (Blyth et al., 1998), while another meta-analysis shows that COPD patients treated with ICS display shows reduced CD8^+^ and CD4^+^ in bronchial biopsies and decreased lymphocyte and neutrophils counts in BALF (Jen et al., 2012). Interestingly, ICS discontinuation in COPD patients results in increased bronchial CD3^+^, CD4^+^, and CD8^+^, along with increased sputum total cell counts (Kunz et al., 2017), further demonstrating the local anti-inflammatory effect of ICS.

Inhaled bronchodilators used in asthma, COPD, and CF principally include β_2_-agonists and antimuscarinics, whose purpose is to widen the lower airway caliber by inducing smooth muscle cell relaxation. Short- and long-acting molecules have been developed for both therapeutic classes, finally delimiting four bronchodilators families: short- and long-acting β_2_-agonists (SABA and LABA) and short- and long-acting antimuscarinics (SAMA and LAMA). A list of available molecules is provided in Table 2.

**TABLE 2:** Non-exhaustive list of bronchodilator molecules used in the treatment of chronic obstructive respiratory disorders and corresponding therapeutic class.

Therapeutic class	Available molecules
SABA	Salbutamol, terbutaline, fenoterol
SAMA	Ipratropium
LABA	Formoterol, indacaterol, olodaterol, salmeterol, vilanterol
LAMA	Aclidinium, glycopyrronium, tiotropium, umeclidinium

β*-Agonists.* In Calu-3 cells cultured in ALI, salmeterol exposure increases TER. This decreased permeability results in slower transepithelial transport of fluticasone propionate (a usual ICS), potentially prolonging ICS anti-inflammatory effects at the AE level (Haghi et al., 2013). Intriguingly, counterintuitive results were obtained by exposing IL13-primed mice either to formoterol or to β-blocker nadolol. Thus, formoterol enhanced IL13-induced asthma features, while nadolol reduced them, suggesting a role for β_2_-adrenergic signaling in the development of asthma epithelial abnormalities (Nguyen et al., 2017), as corroborated in ovalbumin-challenged mice displaying worsened airway hyperresponsiveness (Riesenfeld et al., 2010). Accordingly, formoterol and salmeterol stimulation of primary tracheal epithelial cells and BEAS-2B cells resulted in increased IL-6 and IL-8 release (Strandberg et al., 2007; Ritchie et al., 2018). In the 1980s, several studies also showed that β_2_-agonists increase ciliary beating, both by increasing intracellular cyclic AMP levels and a more complex prolonged calcium-independent mechanism (Wong et al., 1988a, b; Sanderson and Dirksen, 1989; Di Benedetto et al., 1991; Devalia et al., 1992; Frohock et al., 2002), and seems to induce mucus secretion (at least in cat and ferret studies) following cyclic AMP and PKA-dependent mechanisms (Liedtke et al., 1983; Kyle et al., 1988).

*Antimuscarinics.* Tiotropium has long been the only available LAMA, and its effects on the AE have therefore been more largely explored. First, tiotropium has been to shown to reduce AE inflammation, as it downregulates NF-kB activation and decreases IL-1β and IL-6 cytokine production following rhinovirus 14 infection of human tracheal epithelial cells (Yamaya et al., 2012). Similarly, tiotropium-treated BEAS 2B cells exhibit decreased LPS-induced IL-8/CXCL-8 secretion (Suzaki et al., 2011), while tiotropium also reduced viral replication *in vitro* in RSV-infected HEp-2 cells (Iesato et al., 2008). In CS-exposed 16HBE cells, tiotropium prevents the proinflammatory effects of CS, including ERK1/2-related leukotriene release (Profita et al., 2011). In ALI-HBEC from healthy donors, tiotropium does not influence epithelial cell differentiation but is able to reverse goblet cell hyperplasia, probably in a FOXA2/3-dependent manner (Kistemaker et al., 2015). In addition, control BEAS-2B cells exposed to CS alone exhibit cell death and autophagy but are protected from CS deleterious effect upon tiotropium/olodaterol treatment (Chen et al., 2020). Taken together, these data highlight a protective potential effect of tiotropium on the AE, especially by regulating inflammatory mechanisms triggered by external aggressions.

## Discussion and Conclusion

The epithelium represents a key tissue in the lung that stores and coordinates several components of protection against external, inhaled agents. It is also a key target tissue where several chronic diseases have their root and trigger their pathogenic processes. Irrespectively of the genetic or acquired nature of the disorder, several aspects of epithelial integrity may become abnormal, subsequently contributing to further promote disease through vicious circles of repeated harmful challenge, altered frontline defense, chronic/recurrent infection, and uncontrolled inflammation. Altered apico-basal polarization with disturbed junctional protein expression or location or decreased basal-to-apical transcytosis of Igs represents generic features of complex airway diseases such as asthma, COPD, or CF. In addition, mucus hypersecretion and altered mucociliary clearance of inhaled particles and pathogens are aggravated by underlying alterations in differentiation patterns of basal cells that lead to reduced ciliated cell numbers and goblet cell hyperplasia as well as squamous metaplasia in COPD. The mechanisms leading to those changes are complex and include aberrant responses to inhaled agents (allergens, CS, microorganisms) through signaling pathways that connect the epithelium to the immune system or to structural repair. Furthermore, the interplay between the host epithelium and surrounding mesenchyme and immune cells, on the one hand, and the microbiome, on the other hand, plays a previously underestimated role in shaping the epithelial barrier, where dysregulation may contribute at several levels to disease pathogenesis. Future research should better delineate how and to what extent the epithelial barrier is imprinted by genetic and epigenetic marks in order to define innovative strategies aiming at restoring mucosal barrier homeostasis.

## Author Contributions

FC and CDF wrote the manuscript. CP wrote and revised the manuscript. All authors contributed to the article and approved the submitted version.

## Conflict of Interest

The authors declare that the research was conducted in the absence of any commercial or financial relationships that could be construed as a potential conflict of interest.

## Abbreviations

α-SMA: alpha smooth muscle actin
AE: airway epithelium
AEC: alveolar epithelial cells
AJC: apical junctional complexes
ALI: air/liquid interface
AMP: adenosine monophosphate
AREG: amphiregulin
ASL: airway surface liquid
ATP: adenosine triphosphate
BAFF: B-cell activating factor
BALF: bronchoalveolar lavage fluid
CDHR3: cadherin-related family member 3
CF: cystic fibrosis
CFTR: cystic fibrosis transmembrane regulator
COPD: chronic obstructive pulmonary disease
CS: cigarette smoke
CSF1: colony-stimulating factor 1
CXCL: C-X -C motif chemokine ligand
DAMP: damage-associated molecular pattern
DNA: deoxyribonucleic acid
ds: double-strand
ECM: extracellular matrix
EGF: epidermal growth factor
EGFR: epidermal growth factor receptor
EMT: epithelial-to-mesenchymal transition
EMTU: epithelial–mesenchymal trophic unit
ERK: extracellular signal-regulated kinase
GM-CSF: granulocyte-macrophage colony-stimulating factor
GRO-α: growth-regulated oncogene alpha
HBEC: human bronchial epithelial cells
HDM: house dust mite
HIF: hypoxia-inducible factor
HSP: heat shock protein
IFN: interferon
Ig: immunoglobulin
IL: interleukin
IPF: idiopathic pulmonary fibrosis
JAM: junctional adhesive molecules
KLF4: Kruppel-like factor 4
LPS: lipopolysaccharide
MAL: myelin and lymphocyte protein
MAPK: mitogen-activated protein kinase
MARVEL: MAL and related proteins for vesicle trafficking and membrane link
MCP-1: monocyte chemoattractant protein 1
MMP: matrix metalloproteinase
NADPH: nicotinamide adenine dinucleotide phosphate
NET: neutrophil extracellular trap
NICD3: Notch 3 intracellular domain
NLR: NOD-like receptor
NLRX1: NLR family member X1
OCLN: occludin
PA: *Pseudomonas aeruginosa*
PAMP: pathogen-associated molecular pattern
pIgR: polymeric immunoglobulin receptor
PKC: protein kinase C
PLC: phospholipase C
PM: particulate matter
PRR: pattern recognition receptor
RNA: ribonucleic acid
ROS: reactive oxygen species
RSV: respiratory syncytial virus
RV: rhinovirus
SA: *Staphylococcus aureus*
SC: secretory component
SCGB1A1: secretoglobulin family 1A member 1
S-IgA: secretory immunoglobulin A
SLPI: secretory leukoprotease inhibitor
TER: transepithelial electrical resistance
TGF-β: transforming growth factor beta
TJ: tight junctions
TNF-α: tumor necrosis factor alpha
T_*RM*_: tissue-resident memory T cells
TSLP: thymic stromal lymphopoietin
TWIST1: Twist Family BHLH transcription factor 1
UIP: usual interstitial pneumonia
WNT: Wingless/Integrase-1
ZO: zonula occludens.
